# Cross-talk between nervous, immune, and metabolic systems

**DOI:** 10.1186/s41232-022-00222-w

**Published:** 2022-12-03

**Authors:** Yoshimitsu Nakanishi, Sujin Kang, Atsushi Kumanogoh

**Affiliations:** 1 Department of Respiratory Medicine and Clinical Immunology, Graduate School of Medicine, Osaka University, Suita City, Osaka 565-0871 Japan; 2 Department of Immunopathology, Immunology Frontier Research Center, Osaka University, Suita City, Osaka 565-0871 Japan; 3 Integrated Frontier Research for Medical Science Division, Institute for Open and Transdisciplinary Research Initiatives (OTRI), Osaka University, Suita City, Osaka 565-0871 Japan; 4 Department of Advanced Clinical and Translational Immunology, Graduate School of Medicine, Osaka University, Suita City, Osaka 565-0871 Japan; 5 Department of Immune Regulation, Immunology Frontier Research Center, Osaka University, Suita City, Osaka 565-0871 Japan; 6 Center for Infectious Diseases for Education and Research (CiDER), Osaka University, Suita, Osaka 565-0871 Japan

Recent studies have revealed essential roles of the immune system in the development of a variety of metabolic and nervous system disorders, establishing a new dogma that mutual interactions between nervous, immune, and metabolic systems are pivotal for maintaining physiological homeostasis. The molecular mechanisms integrating neural, immune, and metabolic responses are receiving increasing attention and are subjects of intense research.

In this thematic review series entitled “Cross-talk between nervous, immune, and metabolic systems,” we have invited three leading research groups to review immunological studies from the perspective of clinical implications in metabolic and neuronal diseases.

In the first review, Dr. Suganami and colleagues from Nagoya University delve into innate immune responses in the development of metabolic syndrome, with a particular focus on two innate immune receptors, Toll-like receptor 4 (TLR4) and macrophage-inducible C-type lectin (Mincle). Adipocyte-derived saturated fatty acids in adipose tissue stimulate TLR4 signaling in macrophages, leading to chronic inflammation and obesity. In addition to TLR4, other innate immune receptors are also involved in the development of metabolic diseases. In particular, Mincle plays a key role in adipose tissue remodeling.

In the second review, our group from Osaka University summarized immune–metabolic cross-talk from the perspective of immune modulation by axon guidance cues, highlighting its clinical implications in metabolic diseases, such as obesity, diabetes, and atherosclerosis. Semaphorins function not only as axon guidance cues but also as immune modulators, thereby exerting multifaceted effects in the nervous, immune, and metabolic systems. Although semaphorin-mediated coupling of these systems is largely undemonstrated, accumulating evidence suggests that semaphorin signaling is a promising therapeutic target for immunometabolic diseases.

In the third review, Drs. Itokazu, Yamashita, and their colleagues from Osaka University reviewed the essential roles of microglia, which are resident immune cells of the central nervous system, in regulating chronic pain caused by neuroplasticity. Microglial regulation of chronic pain is multi-layered. Microglia regulate synaptic remodeling and modify neural circuits by physically interacting with neurons and by releasing humoral factors essential for neural development and activity.

Here, we would like to express our sincere appreciation to the distinguished researchers who contributed to this theoretical review series, and we hope that these review articles will provide novel insights to researchers in the broad field of inflammation and regeneration.

